# Medical advice-seeking behaviors based on transaction cost theory

**DOI:** 10.1186/s12962-018-0167-y

**Published:** 2018-12-14

**Authors:** Shih Han Lin, Tom M. Y. Lin

**Affiliations:** No. 3 Keelung Road Sec. 4, Taipei, Taiwan

**Keywords:** Transaction cost, Medical advice-seeking behavior, Medical decision-making

## Abstract

**Background:**

Given the global trend of aging societies, medical expenditure has hit record highs in many countries. Because medical advice-seeking behaviors can affect the health of whole societies, how members of a society make medical-related decisions with limited available resources is worthy of investigation. Although transaction cost theory has been extensively applied in commercial research, it is yet to be applied in studies on medical advice-seeking behaviors.

**Method and results:**

This study conducted in-depth interviews with 15 participants and verified that transaction cost theory is applicable for analyzing people’s medical advice-seeking behaviors.

**Conclusion:**

This study verified that transaction cost theory influenced the participants’ choices of physicians and treatment methods, which implies that improved transparency of medical information could considerably reduce transaction costs in relation to medical behaviors and enhance people’s well-being.

## Background

Population aging is an international phenomenon revealing a steadily increasing life expectancy among geriatric people. With the most rapidly aging society worldwide, Taiwan is poised to become an aged society. According to statistics from the Executive Yuan, the aged population in Taiwan will reach 4.75 million by 2025, denoting that one in every five citizens will be over 65 years of age. The status of Taiwan’s society as aging is aggravated by the increasing occurrence of chronic diseases among the geriatric population. Shortages of medical resources have prevented large numbers of older adults with chronic diseases from receiving proper medical attention and treatment, thereby leading to numerous acute complications and consequential medical consumption behaviors, which have caused not only considerable consumption of scarce medical resources but also a decline in medical care quality that has induced numerous problems [6].

The idea and importance of transaction costs was initially described by Ronald Coase in 1937. In 1975, Oliver E. Williamson developed a comprehensive framework for transaction cost theory based on Coase and related literature. Transaction cost theory is a principal theory for management and organization studies [4].

Medical advice-seeking behaviors can influence the prognosis of a disease, medical expenses, and equity in access to medical resources. “Transactions” involving medical services constitute critical decisions many people must make in their lives. Decisions or “transactions” involving choices of food, clothing, education, and jobs broadly and deeply influence human lives.

Although most related studies have concentrated on medical advice-seeking behaviors in relation to various diseases or variations in medical advice-seeking behaviors between different societal groups, the present study, used the transaction cost theory to deconstruct medical advice-seeking behaviors from the standpoint of the users (Tables 1, 2).

**Table 1:** Participant information

Codename	Age	Monthly income NT$/US$	Occupation
1	22	9000/300	University student
2	22	12,000/400	University student
3	23	15,000/500	University student
4	22	13,000/433	University student
5	22	10,000/333	University student
6	22	10,000/333	University student
7	22	10,000/333	University student
8	21	25,000/833	University student
9	21	15,000/500	University student
10	21	12,000/400	University student
11	22	6000/200	University student
12	22	10,000/333	University student
13	21	10,000/333	University student
14	23	28,000/933	2-year college salesperson
15	21	8000/266	University student

**Table 2:** Cost types

Construct	Item	Code
Search and information costs	Search costs: monetary	S1
	Search costs: temporal	S2
	Search costs: physical and psychological	S3
Negotiation costs	Negotiation costs: treatment fees	B1
	Negotiation costs: treatment time	B2
	Negotiation costs: transportation time	B3
	Negotiation costs: transportation fees	B4
	Negotiation costs: opportunity costs	B5
Decision-making costs	Decision-making costs: trust	D1
	Decision-making costs: habits	D2
	Decision-making costs: consultations	D3
	Decision-making costs: other medical advice	D4
Policing costs	Policing costs: physical and psychological: concerns over malpractice	M1
	Policing costs: self-initiated online search for treatment and medicine	M2
	Policing costs: seeking a second opinion	M3
Contract execution costs	Contract execution: physical and psychological: harmful consequences of failed treatment	E1
	Contract execution costs: change of treatment	E2
	Contract execution costs: change of physician	E3

## Literature review

Transaction cost theory was introduced by Ronald Coase, a professor at the University of Chicago, in *The Nature of the Firm* published in 1937. Coase questioned the validity of zero transaction cost as described by conventional economic theories and asserted that in the process of a transaction, uncertainty in the environment and the limited rationality of human beings might incur additional costs for the parties involved. Coase termed these costs “transaction costs.” Coase believed that transaction costs referred to the following three types of cost: (1) search cost: the cost of search for required goods; (2) bargaining cost: the cost of negotiating and reaching an agreement; and (3) enforcement cost: the cost of executing a contract and supervising its execution after the transaction has been validated [1].

In 1975, Williamson developed and expanded Coase’s views to promote better awareness of transaction cost theory among economists. He proposed that failure to complete a transaction usually stems from market failure caused by human and environmental factors, which cause difficulties in market transactions and increase transaction costs. The human factors highlighted by Williamson included the bounded rationality and opportunism of human beings, whereas the environmental factors included uncertainty, complexity, and small transaction volumes. In addition to these factors, information asymmetry and the atmosphere were recognized as factors that could increase transaction costs. In 1985, Williamson further proposed that the suppositions of asset specificity and the frequency and uncertainty of transactions could cause organizational failures and increased transaction costs. Therefore, transaction costs were defined as the costs incurred from the transaction process in addition to that agreed by the parties involved. Such costs include those incurred from information searching, negotiating, drafting, maladaption, haggling, setup and running, and bonding [10].

## Research design

### Participants

In the present study, interviews were conducted in October and November 2016 with 15 Taipei-based students all from different regions in Taiwan and various social and economic backgrounds.

### Research method

In-depth interviews were conducted to ensure data completeness and accuracy. Before each interview, the participant was asked to provide consent for notes and an audio recording of the interview to be taken. Upon completion of each interview, the audio recording was transcribed verbatim for further analysis. After each interview had been transcribed in full, the interviewers compiled a memorandum detailing their observations regarding the body language, mood, and speech of the participant as a reference for follow-up research. In addition, to improve precision and for greater reliability and validity, the research team followed the principle of triangulation tests by using multiple sources for evidence, asking the interviewer to examine the transcript, and establishing construct validity [11].

### Data collection and analysis

The research team composed interview guidelines based on the research objectives and conferred with other researchers about the content corresponding to the research questions, which were subsequently used as bases for the interview questions. The qualitative interview relied on the data analysis helix in Creswell et al. [2] to process and analyze the collected data. Data processing in the present study consisted of the following five steps: data management, reading and recording, description, classification, and interpretation. Each interview transcript was processed through data encoding and data classification to determine the research proposition. Data encoding was conducted in one of the following two manners:

1. Based on the interviewed participant and location revealed in the transcript; or
2. Based on the research objectives and constructs determined through the literature review.

## Results and discussion

### Results

#### Transaction costs: search and information costs

Fan [5] emphasized that in real life, information is usually incomplete and there are costs for complete information. Therefore, “incomplete information” and “search and information costs” are causally related. In transactions and negotiations, all parties attempt to maximize their profits by gaining sufficient information, which costs considerable time, energy, and money, or in other words, transaction costs. Solomon [9] suggested that consumers facing consumption-related problems require assistance from related information for the decision-making process and the search for such information could be referred to as search costs.

*#1:41-50:S2* (Participant 1 discussing the temporal costs of searching and gathering information in lines 41–50 of the transcript)

“So how much time would you spend to make sure the information is correct?”

“About half an hour.”

“You’d spend half an hour checking it and then you would go to the clinic, right?”

“Right.”

*#11:1-3:S3*

“What, in your opinion, are the costs of medical advice-seeking behaviors?”

“Looking up a clinic, registration fees, and waiting times. Before going to see a doctor, I usually prepare myself by making a mental list of my symptoms so that I can readily consult with the doctor and see how I can be cured quickly.”

#### Transaction costs: negotiation costs

Liao [7] proposed that asymmetry in market information coupled with limited rationality and parties engaging in transactions are often unrelated, can foster distrust between the parties that necessitates negotiation, and costs both parties more time, energy, and money. These are negotiation costs.

*#9:1-1:*B2

*“What, in your opinion, are the costs of medical advice-seeking behaviors?”*

*“Registration fees *and* time.”*

*#6:1-1:B3*

“What, in your opinion, are the costs of medical advice-seeking behaviors?”

“Registration fees, transportation time, carfare, and waiting time.”

*#8:3-5:B4*

“Speaking of transportation, if you go to see a doctor by riding a scooter, the cost is the fuel cost, and if you go by mass transportation, then it’s the carfare. Oh, yes, and the registration fee, too.”

*#12:2-6:B5*

“Suppose I have to make an appointment with a doctor. I may have to sacrifice part of my working time or school time. If it’s working time, then it will affect my income. When it comes to money, the costs also include transportation fees and fees for treatment like registration fees.”

#### Transaction costs: decision-making costs

Mutual trust can lower the information searching costs incurred by both parties on acquiring the transaction opportunity. Negotiation costs can be flexibly reduced when both parties are willing to make concessions based on expectations of future gains from each other [8].

*#3:7-11:D1* Decision-making costs: Trust (of people, medical institutions, and treatment)

“So why did you choose that clinic?”

“Because it was close to my place.”

“Locational advantage?”

“Yes.”

“Was there any reason other than locational advantage? Like, maybe you find that doctor trustworthy?”

“That doctor has always been very gentle to patients, which puts you at ease, and he has always been very patient, too.”

“So, simply put, you have grown to trust that doctor.”

“Right, I trust him.”

*#5:18-25:D2* Decision-making costs: Habits

“So you are from Tainan? Do you habitually go to the same clinic or doctor when you are in Tainan?”

“Yes, there is one, and I always go there unless it is not open that day.”

“Is there a reason that you choose this particular clinic or this particular doctor?”

“Because I have been going there since I was little. Also, I find the treatment and the medicine the doctor gives me very effective. Usually, I only have to make one appointment and then I’m cured, unless I’ve got a really bad cold. In that case, I’d usually be cured after the second visit. In addition, I trust that doctor, and there has never been any trouble with him regarding the doctor–patient relationship.”

“So, you are used to that clinic and you trust them?”

“Yes, and I have a very high opinion of them.”

*#14:10-11:D3* Decision-making costs: Consultation (expertise)

“What if you are seriously ill and the doctor at the clinic is unable to cure you?”

“My doctor will refer me to another specialist. He will write a referral letter for me.”

#### Transaction costs: policing costs

Two types of uncertainty are inherent in transactions, both of which can increase policing costs. The first consists of predicable or unpredictable chance events induced by limited rationality and the second is uncertainty in insufficient information rendered by withholding, falsification, or distortion [10].

*#2:70-73:M1*

“So, would you feel worried… I mean, after seeing this doctor, would you still feel worried and seek a second opinion?”

“Yes.”

“Does that mean you still fear the doctor may make an inaccurate diagnosis?”

“Yes. If I am cured with no trouble, then I won’t be worried, but if the sickness persists, I will surely have doubts.”

*#10:68-*74*:M2*

“Would you, for example, search for information online first if you feel very sick and have symptoms that you have never experienced before?”

“Yes, I would search for information on the Internet.”

“What if you find your situation to be kind of different from the doctor’s diagnosis, or even in conflict with it? Which side will you take when that happens?”

“I will show the information to the doctor. I mean, I will show it to him and tell him that it seems to contradict what he said, and then ask him to explain it to me. If his explanation makes sense to me, then of course I will follow his instructions; otherwise, I will go to another clinic for a second opinion.”

*#15:33-40:M3*

“Since you just mentioned that you habitually go to the same clinic whether you are in Taipei or in Miaoli, do you also look for medical advice from other sources?”

“Do you mean the Internet, or any source?”

“Newspaper supplements for example; some people who want to lose weight may follow a diet regime they found in a supplement. Some people with gynecological problems will try traditional Chinese medicine to ‘strengthen the body’ in addition to going to a gynecologist. If you don’t have full confidence in this doctor, you may want to get a second opinion from another doctor and compare what they have to say. Do you do such things?”

“I do. I will check what is good for my situation.”

“So you have no problem with traditional Chinese medicine or things like food therapy?”

“That’s right.”

#### Transaction costs: contract execution costs

After a contract has been agreed upon, both parties must verify whether the other has fulfilled the terms of the contract, and in the event of noncompliance, enforce the terms. The costs that ensure the execution of a contract are contract execution costs. Upon completion of a contract, both parties may choose to renew their transaction or one may choose to relinquish their part of the agreement to another partner, in which case, the costs incurred are switching costs [3].

*#1:61-69:E1*

“Would you still feel worried after seeing the doctor? Like, maybe sometimes you will want to see another doctor or conduct an online search of the medicine the doctor prescribed.”

“I’d only worry about the side effects.”

“You mean harm to the body from side effects or failed therapy?”

“Yes.”

“So, in the face of uncertainty, would you look up information on the medicine prescribed or maybe even stop taking it altogether?”

“I think I would visit another doctor.”

“Do you mean you would counter the uncertainty by changing to another doctor?”

*#7:81-87:E2*

“What would you do if it turned out that the mole removal had failed?”

“I think I would be very angry.”

“What else?”

“If they dare ask me to give them a rating, I’d give them the worst one and demand compensation.”

“Would you go back to them for remedial measures?”

“No. I’d change to another clinic.”

“You’d switch clinic?”

“Of course, it’s for my face after all.”

“Suppose the original clinic offers method A for mole removal and the clinic you intend to switch to offers method B. Would you still want to switch to the new method?”

“I suppose I would. The old method has failed anyway, so why not give another method a try? Yes, I would try it. I would be open to anything that could solve the problem.”

*#3:39-44:E3*

“Would you be worried if the doctor asked you whether you are allergic to anything or feel uncomfortable about taking certain medicines? Would you perhaps worry that the doctor may overlook certain small things at your expense; for example, giving you unsuitable medicine without making sure you aren’t allergic to it?”

“Not at all.”

“Would you change therapy if you felt uncomfortable after taking the medicine? Would you switch treatments or even switch to another clinic?”

“Yes, if that happened I would.”

### Discussion

The interview transcripts indicate that there is a tight relationship between transaction cost theory and medical-seeking behavior. Although, the public has only a weak conception of transaction costs. The transcripts, traces of understanding of transaction costs can be detected in relation to medical advice-seeking behaviors. Most of the discernable elements corroborate the findings of Dahlman [3], including search and information costs, negotiation costs, policing costs, and contract execution costs.

#### Transaction assets

In addition to transaction costs, another factor tentatively referred to as transaction assets has been mentioned repeatedly. This factor may be accumulated at the expense of multiple costs and may facilitate the reduction of future costs. The possession of transaction assets can effectively reduce transaction costs and such assets may be the result of trust, habits, and connections. Experienced people tend to rely on such assets to effectively reduce transaction costs, or in other words, obtain access to equal or preferential medical treatment; for example, some participants stated that they need not spend much time searching for clinics when at home because they are already acquainted with local physicians. However, after leaving home for further education or work, people must spend more time or use connections to build up trust. Therefore, transaction assets can be understood as reducing search and information costs, negotiation costs, policing costs, and contract execution costs.

#### Minor and serious illnesses

Generally, when faced with familiar symptoms, people tend to seek medical advice from medical institutions that incur lower transaction costs such as those that are closer to home, charge less, or do not have long waiting times. By contrast, when faced with unfamiliar symptoms, people feel inclined to incur higher search and information and negotiation costs to prevent the illness from developing beyond their control, thereby ultimately incurring higher policing and contract execution costs.

## Conclusions and suggestions

The present study employed transaction cost theory to analyze considerations in medical advice-seeking behaviors from the perspective of patients. The following conclusions were drawn from the in-depth interviews: transaction costs play a critical role in the selection and decision-making processes inherent in medical advice-seeking behaviors. Although search and information costs may initially appear substantial, the acquisition of accurate information can considerably reduce follow-up transportation times, transportation fees, policing costs, and contract execution costs for failed treatment.

After discovering the relationship between medical-seeking behavior and transaction cost theory, we can understand the concern and the logic of patient when seeking medical treatment. This understanding can serve as the foundation for the healthcare provider and government when caring patient and the public. For example, the government can implicate family medicine policy, and by having a trustworthy physician close to patient's house, mean to help the patient to acquire a transaction asset that reduces future transaction cost.

In addition to verifying the role of transaction costs in medical advice-seeking behaviors, this study detected a number of behaviors extended from transaction cost considerations, the most notable of which being (1) the inclination to accumulate transaction assets such as trust to reduce future transaction costs; and (2) reliance on personal experience to determine the severity of an illness and transaction costs to be invested.

Except for the aforementioned findings, the limited sample size prevented this study from addressing other questions such as how various societal groups recognize transaction costs or the methods adopted by groups to reduce transaction costs. Such questions require further study.

## References

[1] Coase RH (1937). The nature of the firm. Economica.

[2] Creswell JW, Plano Clark VL, Gutmann ML, Hanson WE, Tashakkori A, Teddlie C (2003). Advanced mixed methods research designs. Handbook on mixed methods in the behavioral and social sciences.

[3] Dahlman CJ (1979). The problem of externality. J Law Econ.

[4] David RJ, Han SK (2004). A systematic assessment of the empirical support for transaction cost economics. Strat Manag J.

[5] Fan SP. Political and economic factors in the foreign trade system of mainland China: an analysis applying transaction cost theory. 1998.

[6] Huang PL (2016). Establishment of an integrated intelligent Health Care Network: a case study of the medical steward project in Zhongli.

[7] Liao CH (2009). Listed companies issue securities and trade on-line directly in transaction cost theory.

[8] Liu FW, Li L, Xue YK (2009). Trust, transaction cost and mode of trade credit. Econ Res J.

[9] Solomon MR (2014). Consumer behavior: buying, having, and being.

[10] Williamson OE (1985). The economic intstitutions of capitalism.

[11] Yin RK (2011). Applications of case study research.

